# Comprehensive bioinformatics analysis reveals biomarkers of DNA methylation-related genes in varicose veins

**DOI:** 10.3389/fgene.2022.1013803

**Published:** 2022-11-25

**Authors:** Shengyu Li, Yuehan Liu, Mingming Liu, Lizhao Wang, Xiaofeng Li

**Affiliations:** ^1^ Department of Vascular Surgery, Tianjin First Central Hospital, Tianjin, China; ^2^ Department of Functional Examination, Beijing Aerospace General Hospital, Beijing, China

**Keywords:** varicose veins, DNA methylation, machine learning, biomarkers, bioinformatics

## Abstract

**Background:** Patients with Varicose veins (VV) show no obvious symptoms in the early stages, and it is a common and frequent clinical condition. DNA methylation plays a key role in VV by regulating gene expression. However, the molecular mechanism underlying methylation regulation in VV remains unclear.

**Methods:** The mRNA and methylation data of VV and normal samples were obtained from the Gene Expression Omnibus (GEO) database. Methylation-Regulated Genes (MRGs) between VV and normal samples were crossed with VV-associated genes (VVGs) obtained by weighted gene co-expression network analysis (WGCNA) to obtain VV-associated MRGs (VV-MRGs). Their ability to predict disease was assessed using receiver operating characteristic (ROC) curves. Biomarkers were then screened using a random forest model (RF), support vector machine model (SVM), and generalized linear model (GLM). Next, gene set enrichment analysis (GSEA) was performed to explore the functions of biomarkers. Furthermore, we also predicted their drug targets, and constructed a competing endogenous RNAs (ceRNA) network and a drug target network. Finally, we verified their mRNA expression using quantitative real-time polymerase chain reaction (qRT-PCR).

**Results:** Total three VV-MRGs, namely Wnt1-inducible signaling pathway protein 2 (WISP2), Cysteine-rich intestinal protein 1 (CRIP1), and Odd-skipped related 1 (OSR1) were identified by VVGs and MRGs overlapping. The area under the curves (AUCs) of the ROC curves for these three VV-MRGs were greater than 0.8. RF was confirmed as the optimal diagnostic model, and WISP2, CRIP1, and OSR1 were regarded as biomarkers. GSEA showed that WISP2, CRIP1, and OSR1 were associated with oxidative phosphorylation, extracellular matrix (ECM), and respiratory system functions. Furthermore, we found that lncRNA MIR17HG can regulate OSR1 by binding to hsa-miR-21-5p and that PAX2 might treat VV by targeting OSR1. Finally, qRT-PCR results showed that the mRNA expression of the three genes was consistent with the results of the datasets.

**Conclusion:** This study identified WISP2, CRIP1, and OSR1 as biomarkers of VV through comprehensive bioinformatics analysis, and preliminary explored the DNA methylation-related molecular mechanism in VV, which might be important for VV diagnosis and exploration of potential molecular mechanisms.

## Introduction

Varicose veins (VV) are a common manifestation of chronic venous disease, commonly affecting the lower extremities, causing twisted and dilated veins (Fukaya et al., 2018; Raetz et al., 2019). Approximately 25% of adults suffer from varicose veins (Li et al., 2014). VV can cause systemic symptoms, such as aching, heaviness, cramping, throbbing, restlessness, and swelling in the legs (Gloviczki et al., 2011). The exact pathophysiological mechanism of VV remains controversial but might be related to genetic predispositions, defects in the valves, weakening of the vascular walls, and high intravenous pressure (Raetz et al., 2019). VV is sometimes dismissed as a mere cosmetic issue, but in 20% of patients, it can lead to life-threatening ulcers (Beebe-Dimmer et al., 2005). According to recent studies, patients with VV are up to 5-fold more likely to develop deep vein thrombosis. In addition, it may lead to peripheral arterial disease (Chang et al., 2018). There is an economic burden on the society because varicose diseases have serious consequences. In the United States, more than 30 million adults suffer from VV, resulting in direct medical costs of over $ 1 billion annually (Gloviczki et al., 2011). Therefore, it is important to understand the molecular mechanism underlying VV and to identify potential biomarkers and drug targets that are likely to be effective in preventing it.

DNA methylation is the most prevalent DNA modification and plays an important role in both eukaryotic and prokaryotic life processes (Yang et al., 2020). As an epigenetic mechanism, it can influence gene expression through processes that affect DNA structure and stability, and chromatin structure (Kundu and Peterson, 2009; Jiang et al., 2014). Furthermore, DNA methylation is the main epigenetic form of gene expression regulation in mammals (Huang et al., 2015), mostly occurring on CpG islands in the gene promoter region, where methylation can repress gene transcription (Qin et al., 2015). Studies have shown that DNA methylation status might be involved in the regulation of atherogenesis, diabetes, inflammation, and hypertension (Friso et al., 2008; Ling and Groop, 2009; Turunen et al., 2009). Smetanina et al. (2018) delivered new viewpoints that MFAP5 might play an important role in VV as a master regulator. At meantime, the study provided a preliminary evidence that DNA methylation critically effected on the initiation and progression of VV through function enrichment and systems biology analyses. It was the first study combining OMIC data, including transcriptomics and methylation data, to study the mechanism of VV pathogenesis. Based on the results, we further explored the DNA methylation-related molecular mechanism in VV with same datasets using comprehensive bioinformatics analysis. Besides, whole transcriptome data from 45 samples in GSE36809 dataset were first extacted to analyze the relationship between biomarkers and related miRNA, lncRNA in VV (Xiao et al., 2011; Seok, 2015).

Machine learning can train computers to perform tasks by identifying patterns in massive datasets to determine the rules or algorithms that optimize task completion (Lynch and Liston, 2018). Machine learning has been reported to predict secondary structural features of proteins (Wang et al., 2021) and facilitate the discovery of biomarkers for predicting diseases (Kourou et al., 2015; Lynch and Liston, 2018). It is also widely used in the prognosis and diagnosis of a variety of diseases (Wang and Huang, 2011; Kourou et al., 2015), such as in Yang’s research, machine learning can predict the carcinogenic or non-carcinogenic risk genotype of unknown HPV (Yang et al., 2022). Therefore, machine learning can be used to explore biomarkers of VV.

In this study, DNA methylation-related molecular mechanism in VV was investigated for the first time through the comprehensive bioinformatics analyses on transcriptome data, and the biomarkers of VV (WISP2, CRIP1, and OSR1) were identified using three machine learning models. Furthermore, a biomarkers-related lncRNA-miRNA-mRNA-drug network was predicted, which might provide a reference for clinical research and treatment of VV.

## Materials and methods

### Data collection

In this study, mRNA expression and methylation data of VV and normal samples were obtained from the Gene Expression Omnibus (GEO) database (https://www.ncbi.nlm.nih.gov/geo/). The mRNA expression data of seven VV and seven normal samples were obtained from the GSE68309 dataset, while the GSE68319 dataset contained methylation data of seven VV samples and seven normal samples the patient sources for the GSE68319 and GSE68309 datasets were the same. In addition, whole transcriptome data of 45 blood samples were acquired from the GSE36809 dataset.

### Identification of differentially methylated CpGs sites

DMCs were obtained by comparing the methylation sites of VV and normal samples in the GSE68319 dataset using the “ChAMP” (version 2.20.1) R package with a screening condition of *p* < 0.05 (Tian et al., 2017). The “Rideogram” R package (version 0.2.2) was then used to visualize the chromosome annotation information of DMCs (Hao et al., 2020). Finally, we compared the distribution of CpG sites in different regions within genes to obtain differentially methylated regions (DMRs) using the “UpSetR” (version 1.4.0) R package (Conway et al., 2017).

### Functional enrichment analysis of methylation-regulated genes

In the GSE68309 dataset, differentially expressed genes (DEGs) between VV and normal samples were compared using the “edgeR” (version 3.34.1) R package with the screening condition of |log_2_FC| > 0.5, *p* < 0.05 (Robinson et al., 2010). The expression of DEGs was shown by the volcano plot drawn by the “ggplot2” R package (version 3.3.5) (Zhang and Wang, 2022). We then corresponded DMCs to the corresponding genes based on methylation annotation information, and the methylation levels were upregulated for hypermethylated genes and downregulated for demethylated. MRGs were obtained from the intersections of the demethylated and highly expressed genes as well as the hypermethylated and the low-expressed genes. The enrichment analysis of Kyoto Encyclopedia of Genes and Genomes (KEGG) and Gene ontology (GO) was performed using “clusterProfiler” R package (Version 4.0.2) to find the functions and related pathways of the MRGs with the significance threshold of *p* < 0.05 (Yu et al., 2012).

### Identification of diagnostic genes

Weighted gene co-expression network analysis (WGCNA) can cluster modules by gene expression similarity and screen modules with highly correlated traits based on the correlations between modules and traits to find target genes (Langfelder and Horvath, 2008). In this study, we used normal and VV samples as trait data. Total seven normal and seven VV samples in the GSE68309 dataset were used as trait data for WGCNA using the “WGCNA” R package (version 1.70-3) to identify VV-associated genes (VVGs). First, the samples were clustered to determine whether outlier samples needed to be removed. Based on the clinical trait information, sample clustering and clinical trait heat maps were subsequently drawn. To ensure that the interactions between genes maximally conformed to a scale-free distribution, we determined the soft threshold. Based on the optimal soft threshold, we set the minimum number of genes per gene module to 100 and the cutting height to MEDissThres = 0.7, according to the criteria of the hybrid dynamic tree cutting algorithm, to draw the module clustering tree. The modules significantly associated with VV were then identified as key modules based on their correlations with the traits. Finally, scatter plots were drawn for key modules to show the correlations between genes and modules (Module Membership, MM) and between genes and traits (Gene Significance, GS), with the threshold values: |GS| > 0.5, |MM| > 0.7, and *p* ≤ 0.05 to screen VVGs. The VVGs were then intersected with the MRGs to obtain VV-associated MRGs (VV-MRGs). To assess the disease prediction ability of VV-MRGs, receiver operating characteristic (ROC) curve was plotted by the “pROC” (version 1.18.0) R package (Robin et al., 2011). The area under the curve (AUC) value refers to the area under the ROC curve. The larger the value, the more accurate is the prediction. VV-MRGs with AUC values >0.8 were used as diagnostic genes.

### Identification of biomarkers by machine learning algorithms

To screen biomarkers, we calculated the importance of diagnostic genes using three machine learning methods: random forest model (RF), support vector machine model (SVM), and generalized linear model (GLM) (Qu et al., 2022; Wang et al., 2022). The models were analyzed using the “DALEX” R package (version 2.3.0) to plot residual distributions, and algorithmic power box line plots to obtain the best model from which biomarkers were obtained (Floyd et al., 2022). The nomogram of biomarkers was constructed using the “RMS” (version 6.1-0) R package to derive the relationship between biomarkers and diseases, and the calibration curves plotted by “RMS” (version 6.1-0) R package were used to validate the model (Qiu et al., 2022).

### Gene set enrichment analysis of biomarkers

To further investigate the relevant signaling pathways and potential biological mechanisms in VV samples, we used the “clusterProfiler” (version 3.18.1) and the “org.Hs.eg.db” (version 3.12.0) R packages to perform single-gene GO and KEGG enrichment analyses for the diagnostic genes (Li et al., 2021). The median expression values of the diagnostic genes were used to classify the samples into high- and low-risk groups. GSEA enrichment analysis was then performed for all genes, with the threshold set at |NES| > 1, *p* < 0.05, and q < 0.25 (Subramanian et al., 2005).

### Competing endogenous RNA and drug target networks

We downloaded the drug targets from the binding database. We then constructed a protein-protein interaction (PPI) network between drug targets and biomarkers based on the STRING database (von Mering et al., 2003), and selected drug targets with an interaction score >0.7. In order to investigate the relationship for gene expression of biomarkers with targeting miRNA and corresponding lncRNA, the relative information from 45 blood samples within GSE36809 dataset was exteacted. We searched for miRNAs related to biomarkers from the miRwalk and miRDB databases (Liu et al., 2020), merged the results of the two databases, and obtained candidate miRNAs interacting with biomarkers (mRNAs). Next, we searched for miRNAs that crossed with the candidate miRNAs in the GSE36809 dataset, and used the “tidyr” (version 1.1.4) R package to calculate the correlations between biomarkers (mRNAs) and miRNAs (Mangiola et al., 2021). Furthermore, we used miRNAs to predict the lncRNAs in the Starbase database (Li et al., 2014). Finally, mRNA-miRNA and miRNA-lncRNA were obtained based on the above analysis, and a ceRNA network was constructed using Cytoscape software (Shannon et al., 2003). The lncRNA-miRNA-mRNA-drug network was also constructed based on the potential therapeutic drugs identified in the previous step.

### Quantitative real-time polymerase chain reaction validation

Seven patients with VV were recruited from Tianjin First Central Hospital. The study met the ethical requirements of Tianjin First Central Hospital. Samples from diseased and normal venous vessels of VV were taken from the participants, and RNA was extracted using TRIzol Reagent (REF:15596018) provided by Ambion. Reverse transcription was performed using the sweScript RT I First strand cDNA Synthesis All-in-OneTM Kit (REF:15596018) (Servicebio Technology Co., Ltd., in Wuhan, Hubei Province, China). PCR was performed using the 2xUniversal Blue SYBR Green qPCR Master Mix (CAT-G3326-05) kit (Servicebio Technology Co., Ltd., in Wuhan, Hubei Province, China) (Bachman, 2013). The PCR conditions were as follows: pre-denaturation at 95°C for 1 min followed by 40 cycles, each of denaturation at 95°C for 20 s, 55°C for 20 s, and extension at 72°C for 30 s. GAPDH was used as an internal reference for gene detection. Primer sequences are shown in Table 1. The expressions of WISP2, OSR1, and CRIP1 in diseased and normal venous vessels were compared by analysis of variance (ANOVA), and *p* < 0.05 was considered as significant.

**TABLE 1:** Primers used for reverse transcription-quantitative PCR.

Sequence name	Forward primer (5′-3′)	Reverse primer (3′-5′)
WISP2	CTGGATGGCTGTGGCT	AACTGGGGTCCTTGGG
OSR1	AGTGGACGCTGGGCTA	GGGCTTGGGTTGAATG
CRIP1	AAATGTGGGAAGACGCT	GGTGGTTGCAGTAGGGT
GAPDH	CCC​ATC​ACC​ATC​TTC​CAG​G	CAT​CAC​GCC​ACA​GTT​TCC​C

## Results

### Identification of differentially methylated CpGs sites

**FIGURE 1:**
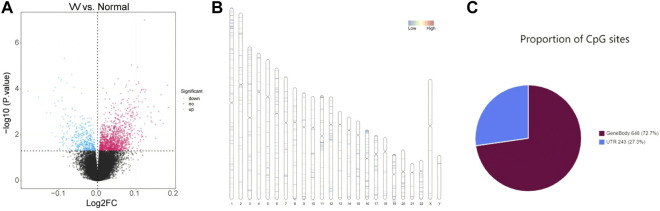
Identification of differentially methylated CpGs sites (DMCs). **(A)** Volcano maps of the DMCs in GSE68319 dataset. **(B)** The locations of DMCs on chromosomes. **(C)** The regional distribution of DMCs in different genes.

The whole flowchart of the study was shown in Supplementary Figure S1. Total 1645 DMCs were screened between VV and normal samples, including 1208 upregulated and 437 downregulated sites, and the results are shown in Supplementary Table S1. Volcano maps were drawn based on the DMCs (Figure 1A). There were 891 CpG sites with annotation information, and Figure 1B shows the locations of the DMCs on the chromosomes. The regional distribution of DMCs in the different genes is shown in Figure 1C. Most (72.7%) of the CpG sites were located within the gene.

### Functional enrichment analysis of methylation-regulated genes

**FIGURE 2:**
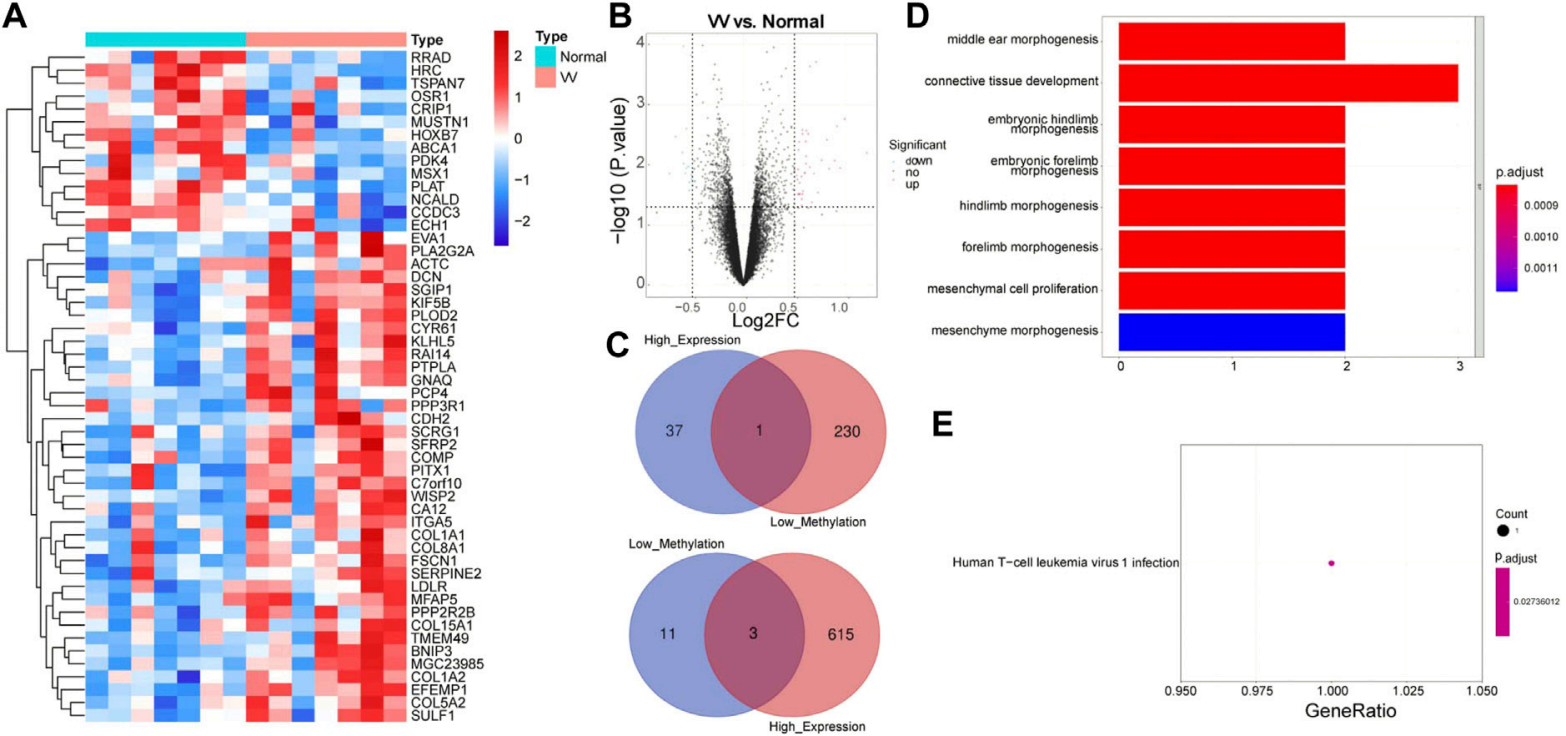
Functional enrichment analysis of Methylation-Regulated Genes (MRGs). **(A)** Heatmap of Differentially expressed genes (DEGs) between VV samples and normal samples in GSE68309 dataset. **(B)** Volcano map of DEGs in GSE68309. **(C)** A Venn diagram of the demethylated genes and the highly expressed genes, and a venn diagram of the hypermethylated genes and the low expressed genes. **(D)** The GO enrichment analysis of MRGs. **(E)** The KEGG analysis enrichment of MRGs.

In the GSE68309 dataset, 52 DEGs were screened out between VV and normal samples, including 38 upregulated and 14 downregulated DEGs (Supplementary Table S2). Volcano and heat maps of DEGs were shown in Figures 2A,B. The demethylated and the highly expressed genes as well as the hypermethylated and the low expressed genes were intersected to obtain four MRGs: WISP2, OSR1, MSX1, and CRIP1, which are shown in Figure 2C. We then annotated the above four MRGs using the KEGG pathway and GO function to explore the biological significance of each gene. A total of 201 GOs and one KEGG pathway were enriched, as shown in Supplementary Table S3. Figure 2D showed the enrichment of the top eight GOs: middle ear morphogenesis, connective tissue development, embryonic hindlimb morphogenesis, embryonic forelimb morphogenesis, hindlimb morphogenesis, forelimb morphogenesis, mesenchymal cell proliferation, and mesenchyme morphogenesis. KEGG enrichment was shown in Figure 2E, and its pathway has been determined to be Human T-cell leukemia virus 1 infection.

### Identification of diagnostic genes

**FIGURE 3:**
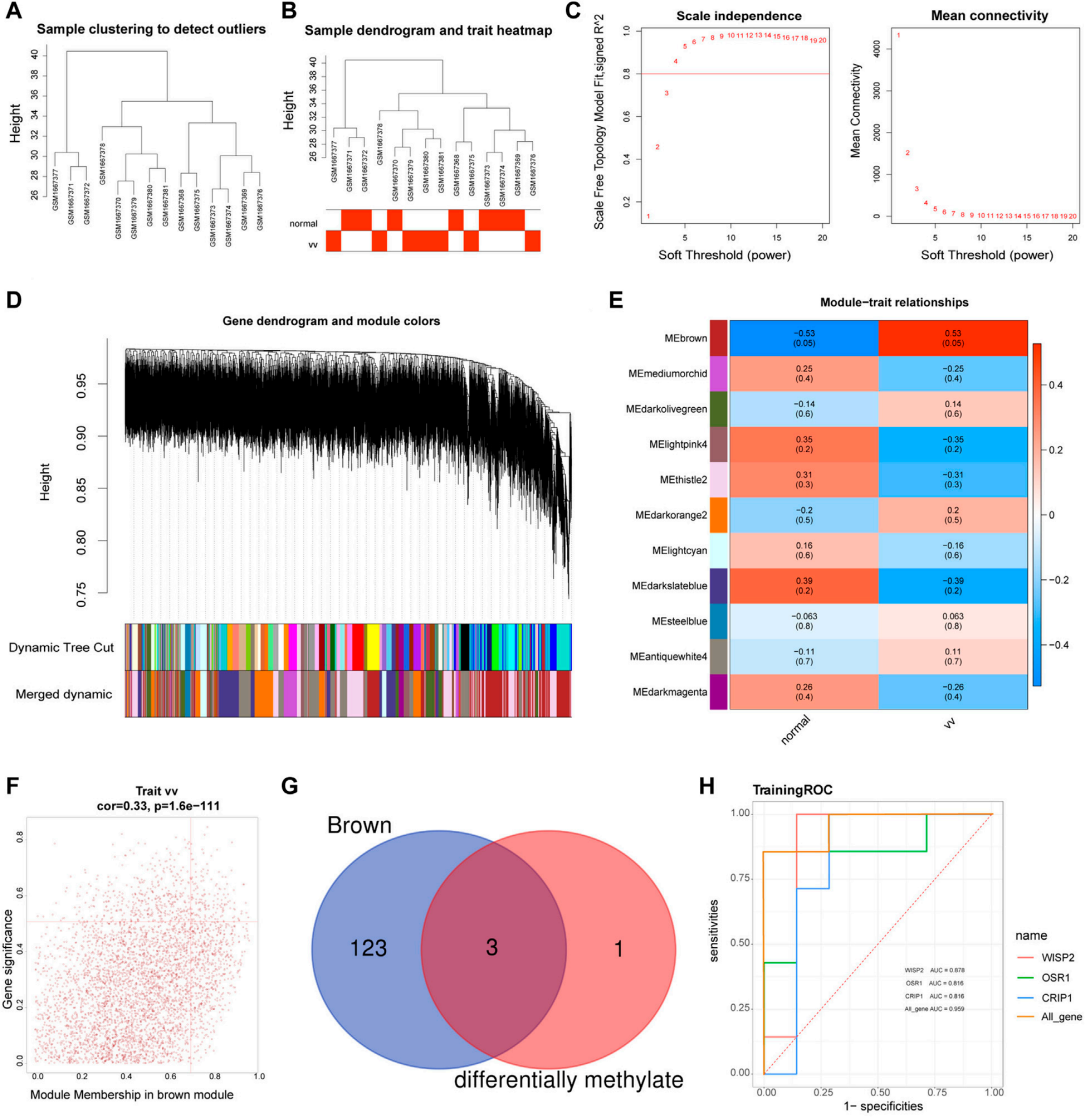
Identification of diagnostic genes. **(A)** The sample clustering plot in the GSE68309. **(B)** Sample dendrogram and sample-trait clustering heatmap. **(C)** Filtering of soft thresholds. The horizontal axis represents the weight parameter β, and the vertical axis on the left is the Scale Free Topology Model Fit. The higher the square of the correlation coefficient is, the closer the network is to the distribution of the scale-free network. The vertical axis of the figure on the right represents the average connectivity of all genes in the corresponding module, which is close to 0 and presents a gentle trend. The network is closer to the scale-free network. **(D)** Module clustering dendrograms. The top half is a hierarchical clustering tree of genes and the bottom half is gene modules. Genes clustered into the same branch are grouped into the same module, with different colours representing different modules. **(E)** Heat map of the relationship between gene modules and traits. Positive correlations are in red, negative correlations in blue. **(F)** MM and GS scatter plots for brown modules. The horizontal coordinates shows the correlation between genes and modules (MM) and the vertical coordinates shows the correlation between genes and traits (GS). **(G)** The Venn diagram of MRGs and brown module genes (VVGs). **(H)** ROC curves of WISP2, CRIP1, and OSR1.

The sample clustering plot showed that there were no outlier samples in the GSE68309 dataset (Figure 3A). The sample clustering and clinical trait heat map showed that the VV and normal samples were clustered well (Figure 3B). The network approximated a scale-free distribution when the power threshold was four (Figure 3C). A total of 23 modules were segmented by the hybrid dynamic shear tree algorithm, and 11 modules were obtained after merging, and the module clustering tree was shown in Figure 3D. The correlations between MODULE and grouped traits were shown in Figure 3E. Among the 11 modules, the brown module (*R* ^2^ = 0.53, *p* = 0.05) had a high and significant positive correlation with VV. Therefore, the brown module was identified as the key module, with18631 genes. The MM and GS scatter plots of the brown module were shown in Figure 3F, and 126 VVGs were obtained (Supplementary Table S4). The intersection of VVGs and MRGs was used to obtain three VV-MRGs: WISP2, CRIP1, and OSR1 (Figure 3G). As shown in Figure 3H, the AUC values for WISP2, CRIP1, and OSR1 were 0.878, 0.816, and 0.816, respectively. Moreover, their combined AUC was 0.905 and displayed excellent diagnostic performance.

### Identification of biomarkers by machine learning algorithms

**FIGURE 4:**
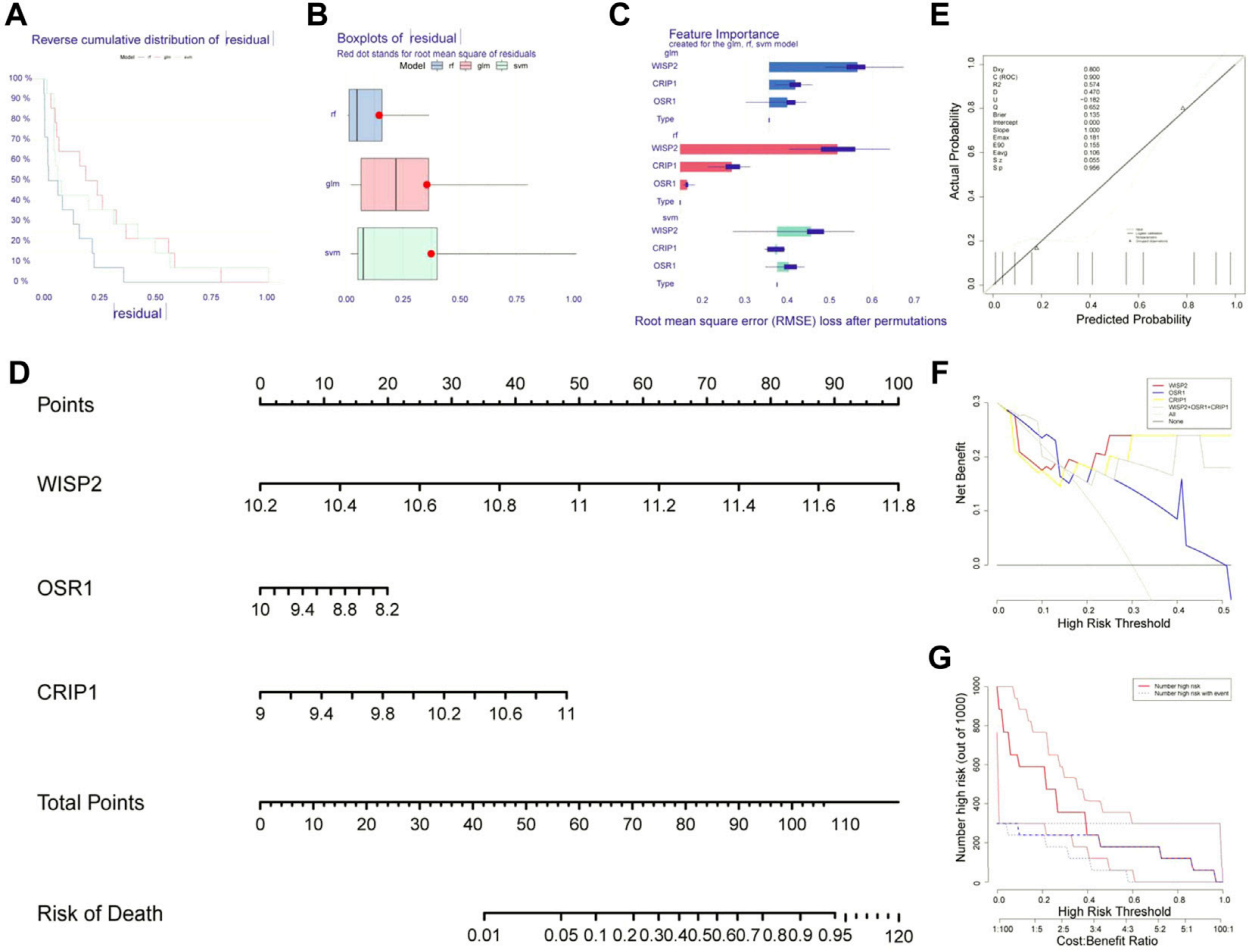
Identification of biomarkers by machine learning algorithms. **(A)** The machine learning residual distribution. **(B)** Algorithm capability box line plots. **(C)** 3 biomarkers WISP2, CRIP1, and OSR1 obtained from the random forest model (RF). **(D)** The nomogram of WISP2, CRIP1, and OSR1. **(E)** The calibration curves of nomogram. **(F)** The decision curve. **(G)** The clinical curves.

The machine learning residual distribution and algorithm capability box line plots were shown in Figures 4A,B, where the RF algorithm outperformed the SVM and GLM algorithms. We then obtained three biomarkers, WISP2, CRIP1, and OSR1, from RF (Figure 4C). The biomarker nomogram was shown in Figure 4D. The calibration curves were plotted in Figure 4E. The results showed that the model passed the calibration degree test with *p* > 0.05, and the ROC area was 0.900 with good discriminatory ability. Overall, the predictive ability of the model was excellent. The decision curve graph showed that the net benefit of all three biomarkers was higher than that of individual biomarkers (Figure 4F). The clinical curves showed that the biomarkers were better predictors of prognosis (Figure 4G).

### Gene set enrichment analysis enrichment analysis of biomarkers

**FIGURE 5:**
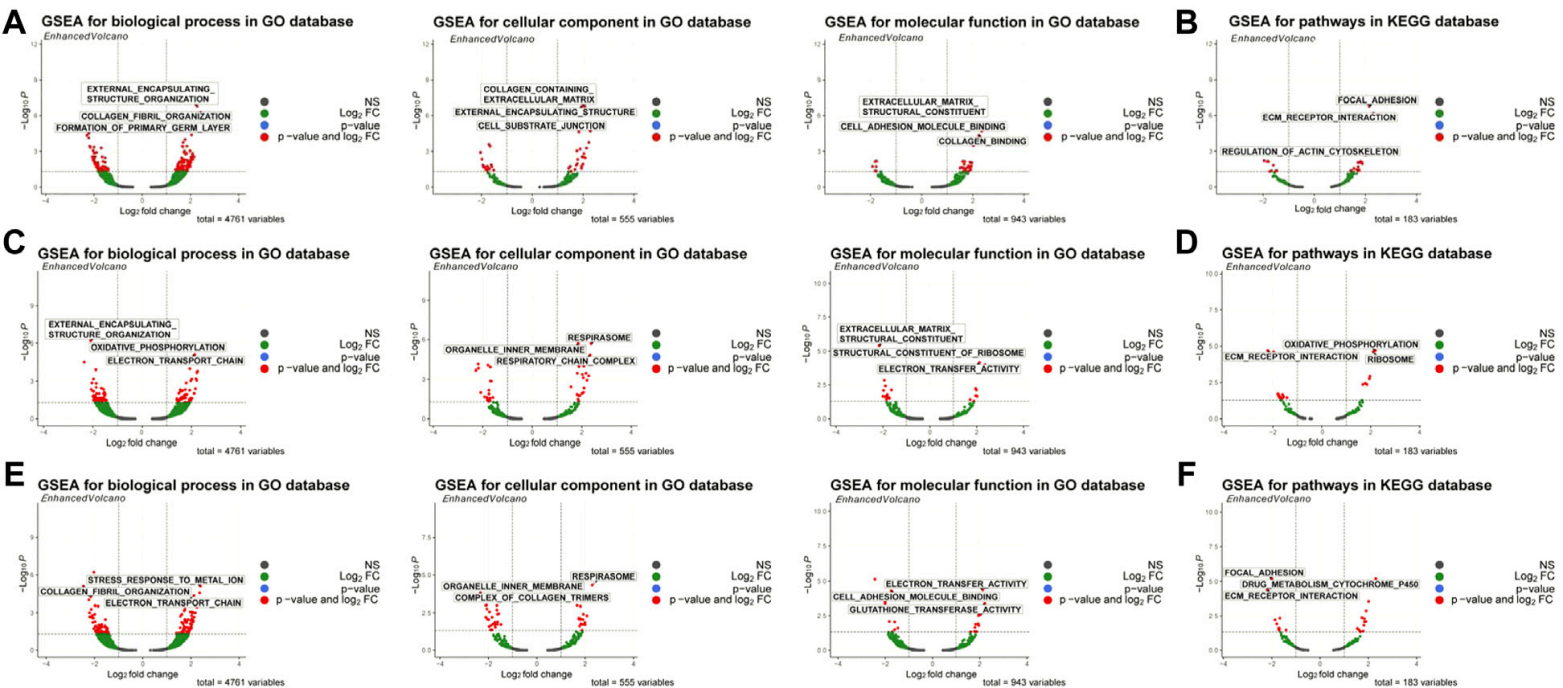
GSEA enrichment analysis of biomarkers. **(A)** GO enrichment ofWISP2. **(B)** KEGG analysis ofWISP2. **(C)** GO enrichment of CRIP1. **(D)** KEGG analysis of CRIP1. **(E)** GO enrichment of OSR1. **(F)** KEGG analysis of OSR1.

First, the WISP2 diagnostic gene was used for single-gene GSEA enrichment analysis, and 262 GO enrichment profiles and 27 KEGG pathways were obtained, including extracellular structure tissue, collagen fibril tissue, collagen-containing ECM and its structural components, focal adhesion, and receptor interactions (Supplementary Tables S5, S6; Figures 5A,B). Single-gene GSEA enrichment analysis was also conducted with the CRIP1 diagnostic gene, and 178 GO enrichment conditions and 21 KEGG pathways were obtained, including external encapsulating structure organization, oxidative phosphorylation, respiratory system, organelle inner membrane, structural components of ECM, structural components of ribosomes, and ECM receptor interactions, etc. (Supplementary Tables S7, S8; Figures 5C,D). OSR1 diagnostic gene was used for single-gene GSEA enrichment analysis, and 206 GO enrichment conditions and 24 KEGG pathways were obtained, including external encapsulating structure organization, collagen fiber organization, cell organelle internal membrane, respiratory system, metabolism of toxic substances by cytochrome p450, ECM structure components that constitute tensile strength, electron transfer activity (Supplementary Tables S9, S10; Figures 5E,F).

### Competing endogenous RNA and drug target networks

**FIGURE 6:**
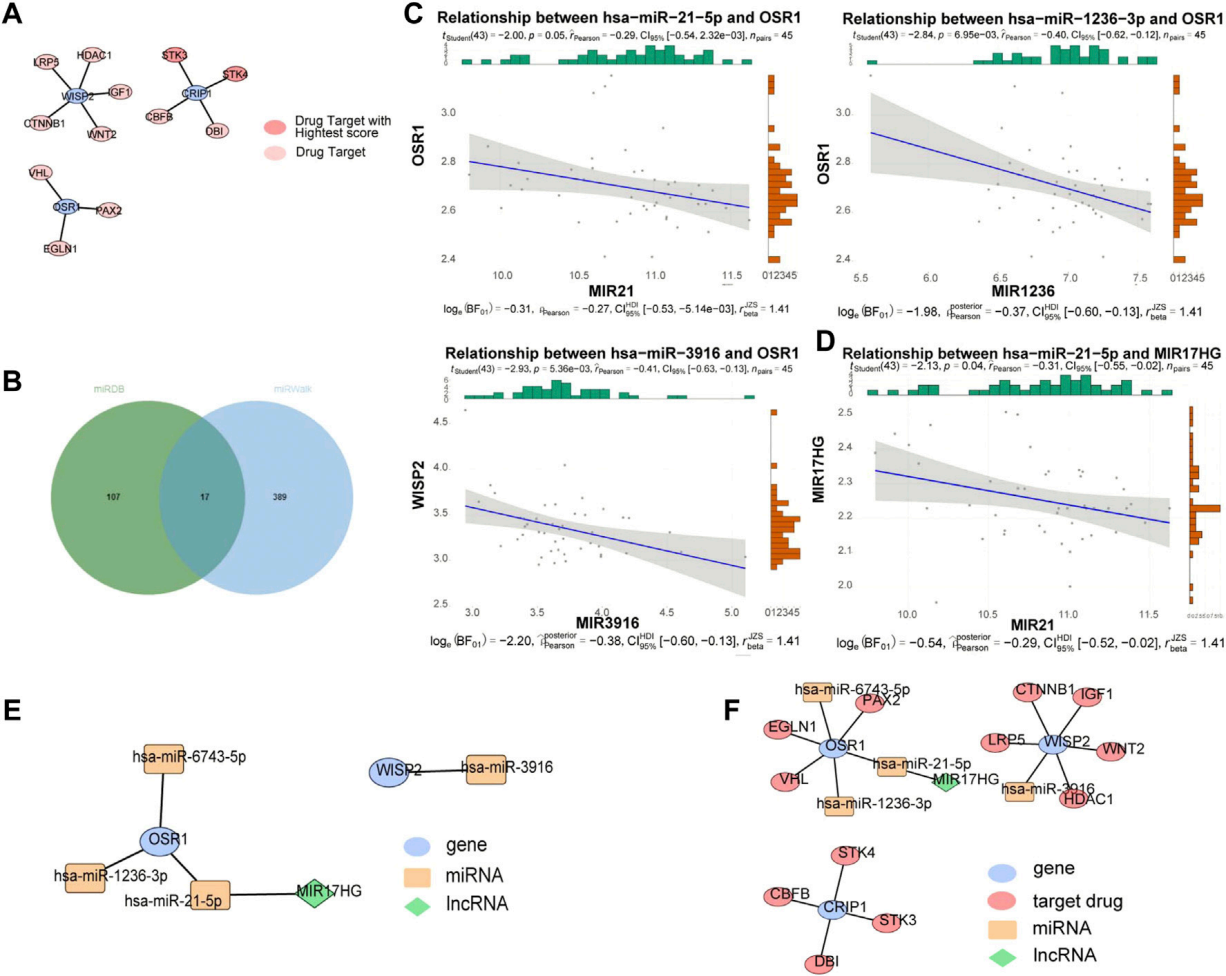
ceRNA and drug targets networks analysis. **(A)** Protein-protein interaction (PPI) network between drug targets and biomarkers based on String database. **(B)** The miRNAs associated with biomarkers in miRwalk and miRDB databases. **(C)** Correlation between OSR1 and 4 miRNAs. **(D)** Correlation between miR-21-5p expression and MIR17HG. **(E)** A ceRNA network. **(F)** A lncRNA-miRNA-mRNA-drug network. Blue ovals: gene; Yellow rectangles: miRNAs; Red ovals: drug targets; Green diamonds: lncRNAs.

A total of 12 drug targets were obtained, of which two were high-scoring targets. Specifically, the WISP2 gene predicted LRP5, HDAC1, IGF1, WNT2, and CTNNB1 drug targets, the CRIP1 gene predicted STK3, STK4, CBFB, and DBI drug targets, and the OSR1 gene predicted VHL, PAX2, and EGLNA drug targets, among which, STK3 and STK4 were high-scoring drug targets (Figure 6A). The miRNAs associated with the biomarkers in the miRwalk and miRDB databases were merged to obtain 513 candidate miRNAs (Figure 6B). We identified 26 miRNAs in the GSE36809 dataset. Based on the ceRNA network, miRNA and mRNA expressions were negatively correlated, and three miRNAs with negative correlations were retained: hsa-miR-21-5p (cor = −0.29, *p* = 0.05), hsa-miR-1236-3p (cor = −0.4, *p* = 0.007), and hsa-miR-3916 (cor = −0.41, *p* = 0.005) were the predicted miRNAs, and the correlation results were shown in Figure 6C. We then used four miRNAs to predict lncRNAs in the starbase database and intersected the results to obtain the lncRNA of hsa-miR-21-5p, and kept one lncRNA with negative correlation: MIR17HG (cor = −0.31, *p* = 0.04) (Figure 6D). The constructed ceRNA network was shown in Figure 6E. The lncRNA-miRNA-mRNA-drug was constructed based on the potential therapeutic drugs identified in the previous step (Figure 6F). We found that lncRNA MIR17HG could regulate OSR1 by binding to hsa-miR-21-5p and that PAX2, EGLN1, and VHL might treat VV by targeting OSR1, CTNNB1, IGF1, LRP5, WNT2, HDAC1 by targeting WISP2, and STK4, CBFB, STK3, and DBI by targeting CRIP1.

### Quantitative real-time polymerase chain reaction validation

**FIGURE 7:**
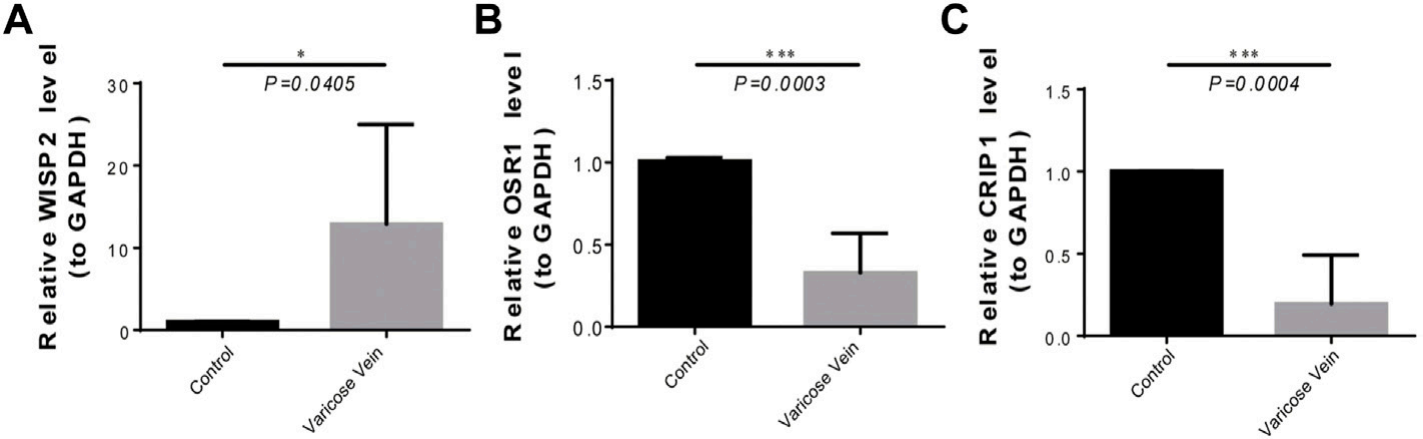
The expression of three biomarkers in qRT-PCR experiments. **(A)** The WISP2 expression in diseased and normal venous vessels. **(B)** The OSR1 expression in diseased and normal venous vessels. **(C)** The CRIP1 expression in diseased and normal venous vessels.

To further validate biomarker expression, we used qRT-PCR to compare the gene expression of WISP2, OSR1, and CRIP1 in diseased and normal venous vessels. The results showed that the expression of the WISP2 gene in diseased vessels was significantly upregulated, and that of OSR1 and CRIP1 genes in diseased vessels was significantly downregulated (Figure 7), as compared to normal samples.

## Discussion

VV is a common peripheral vascular disease presenting twisted and dilated veins, usually in the lower extremities (Hamdan, 2012). Weakening of venous wall, valve dysfunction, and increased intravenous pressure are commonly considered to be its main causes (Hamdan, 2012). VV caused severe underlying vascular insufficiency including eczema, infection, venous ulceration, superficial thrombophlebitis, loss of subcutaneous tissue, and lipodermatosclerosis (Hamdan, 2012; Raffetto et al., 2020). Therefore, early identification of high-risk factors in VV patients and early implementation of targeted preventive interventions will reduce the occurrence of VV and avoid wastage of healthcare resources.

In our study, three VV-MRGs, WISP2, CRIP1, and OSR1, were identified as potential biomarkers. Wnt1-inducible signaling pathway protein 2 (WISP2) is a secreted intracellular protein that is cell-specific and multifunctional (Hammarstedt et al., 2013; Grünberg et al., 2014). That WISP2 has been reported to be possibly regulated by the typical Wnt and TGF-β pathways for treatment of obesity and metabolic diseases, and developing new therapeutic strategies (Grünberg et al., 2018). In addition, it is regulated by the Wnt signaling pathway and IGF-1 to improve the metabolic status of animals (Alami and Liu, 2021). It can promote several types of tumors; for example, ovarian cancer cell proliferation by targeting ERK and YAP (Shi et al., 2020). However, the role of WISP2 in VV has not been previously reported, and we speculate that it may regulate VV through the Wnt signaling pathway. As an intracellular protein, WISP2 has been reported to be an important regulator of BMP4 action.

Cysteine-rich intestinal protein 1 (CRIP1), a LIM protein subfamily, contains a short LIM structural domain (Cai et al., 2017). CRIP1-positive circulating and splenic monocytes have been reported to play an important role in the inflammatory process associated with hypertension, and CRIP1 may influence the interaction of the immune system and the pathogenesis of hypertension (Schweigert et al., 2021). In addition, it can regulate the growth and differentiation of eukaryotic cells (Cai et al., 2017). Therefore, it is hypothesized that CRIP1 regulates VV through the immune system and inflammation.

Odd-skipped related 1 (OSR1) is an odd-skipped family zinc-finger protein (So and Danielian, 1999). OSR1 plays a vital role in heart and urogenital development, such as of kidneys and colon (Wang et al., 2005; Zhang and Jiang, 2020). In our study, it was found to play an important role in VV, suggesting that it can be a novel target for further studies.

GSEA showed that WISP2, CRIP1, and OSR1 are associated with oxidative phosphorylation, ECM, and respiratory system functions. Oxidative phosphorylation maintains homeostasis in all animals and plants (Wilson, 2017), and also plays an important role in vascular endothelial cells (ECs) (Li Q. et al., 2021). ECM remodeling processes regulate VV by venous hypertension (Barallobre-Barreiro et al., 2016). In addition, some respiratory system functions, such as pulmonary embolism (PE) has relationship with VV by venous thromboembolism (VTE) (Otero et al., 2013). Therefore, oxidative phosphorylation, extracellular matrix, and respiratory system might impact VV and provide new directions for further studies on VV.

Drug discovery is a time-consuming, costly, and complex process (Overington et al., 2006; Scannell et al., 2012). Reasonable drug target selection is an efficient strategy to reduce the risk of preclinical drug development (Arrowsmith, 2011; Quan et al., 2018). In addition, promising and potentially high clinical efficacy drug targets have been identified, which is a key step in drug discovery (Dahlin et al., 2015). In this study, 12 drug targets, including IGF-1, VHL, CBFB, and STKK3, were identified by constructing PPI network between drug targets and biomarkers. Among them, it was shown that VHL was associated with elevated vascular endothelial growth factor (VEGF) concentration (Gordeuk et al., 2004). VHL mutations were associated with the development of vascular tumors, such as renal clear cell carcinoma, pheochromocytoma, pancreatic neuroendocrine tumors, and central nervous system hemangioblastoma. Moreover, IGF-1 factor was found to be associated with skin abnormalities, cause localized or diffuse hyperkeratotic plaques with or without hyperpigmentation, and increase the prevalence of VV and psoriasis (Sandru et al., 2021), which is consistent with our study. In addition, STK3 and STK4 are the top two drug targets in predicted drug targets. STK3/4 are important kinases in the Hippo signaling pathway (Cho et al., 2021). Their pharmacological inhibitors are reportedly effective in treatment of acute myeloid leukemia (Bata et al., 2022), and recent data indicate that they are potential therapeutic molecules for suppressing the Hippo signaling pathway, thereby improving tissue repair (Fan et al., 2016). However, their function in VV and contribution to its therapeutic molecules have not been investigated. To the best of our knowledge, CBFB, LRP5, HDAC1, CTNNB1, WNT2, DBI, EGLN1, PAX2, STK3, and STK4 are the first reported drug targets in VV, and further experiments are needed to validate them.

By competitively binding to miRNA response elements (MREs) on mRNA, lncRNAs can cushion and restrain the protein expression of target mRNA, which is called the competitive endogenous RNA (ceRNA) mechanism. Studies have reported that ceRNA and drug target networks can provide effective and innovative therapeutic strategies for diseases. For example, analysis of ceRNA networks and identification of potential drug targets for drug-resistant leukemia cell K562/ADR (Liu et al., 2021), and ceRNA networks provide potential biomarkers and therapeutic targets for colorectal cancer (Wang et al., 2019), and in SARS-CoV-2 infection (COVID-19) (Arora et al., 2020). Therefore, it can provide novel and effective targets for the development of innovative therapeutic strategies against VV. In this study, lncRNA-miRNA-mRNA-drug network was built, and we found that lncRNA MIR17HG can regulate OSR1 by binding to hsa-miR-21-5p, and PAX2 might treat VV by targeting OSR1. Our study identified VV drug targets and their interrelations with miRNAs and lncRNAs; however, further studies are needed to provide new directions for its treatment.

In conclusion, WISP2, CRIP1, and OSR1 are possibly important biomarkers for VV diagnosis, exploration of potential molecular mechanisms, progression, and treatment. However, our study had some limitations. Statistically significant biomarkers were only determined by bioinformatics analysis in this study, but further validation, such as animal experiments and clinical trials is required. Some underlying mechanisms in VV require further investigation, such as gene knockdown and overexpression.

## Data Availability

Publicly available datasets (GSE68309, GSE68319, GSE36809) from GEO database were analyzed in this study. This data can be found in here: https://www.ncbi.nlm.nih.gov/geo/.

## Supplementary material

SUPPLEMENTARY TABLE S1 DMCs between VV samples and normal samples of GSE68319 dataset.

SUPPLEMENTARY TABLE S2 DEGs between VV samples and normal samples of GSE68309 dataset.

SUPPLEMENTARY TABLE S3 Functional enrichment analysis of Methylation-Regulated Genes.

SUPPLEMENTARY TABLE S4 126 VVGs in brown module.

SUPPLEMENTARY TABLE S5 The GO enrichment analysis of WISP2.

SUPPLEMENTARY TABLE S6 The KEGG enrichment analysis of WISP2.

SUPPLEMENTARY TABLE S7 The GO enrichment analysis of CRIP1.

SUPPLEMENTARY TABLE S8 The KEGG enrichment analysis of CRIP1.

SUPPLEMENTARY TABLE S9 The GO enrichment analysis of OSR1.

SUPPLEMENTARY TABLE S10 The KEGG enrichment analysis of OSR1.

## References

[B1] Alami T. Liu J. L. (2021). Metabolic effects of CCN5/WISP2 gene deficiency and transgenic overexpression in mice. Int. J. Mol. Sci. 22 (24), 13418. 10.3390/ijms222413418 34948212 PMC8709456

[B2] Arora S. Singh P. Dohare R. Jha R. Ali Syed M. (2020). Unravelling host-pathogen interactions: ceRNA network in SARS-CoV-2 infection (COVID-19). Gene 762, 145057. 10.1016/j.gene.2020.145057 32805314 PMC7428439

[B3] Arrowsmith J. (2011). Trial watch: Phase II failures: 2008-2010. Nat. Rev. Drug Discov. 10 (5), 328–329. 10.1038/nrd3439 21532551

[B4] Bachman J. (2013). Reverse-transcription PCR (RT-PCR). Methods Enzymol. 530, 67–74. 10.1016/b978-0-12-420037-1.00002-6 24034314

[B5] Barallobre-Barreiro J. Oklu R. Lynch M. Fava M. Baig F. Yin X. (2016). Extracellular matrix remodelling in response to venous hypertension: Proteomics of human varicose veins. Cardiovasc. Res. 110 (3), 419–430. 10.1093/cvr/cvw075 27068509 PMC4872879

[B6] Bata N. Chaikuad A. Bakas N. A. Limpert A. S. Lambert L. J. Sheffler D. J. (2022). Inhibitors of the Hippo pathway kinases STK3/MST2 and STK4/MST1 have utility for the treatment of acute myeloid leukemia. J. Med. Chem. 65 (2), 1352–1369. 10.1021/acs.jmedchem.1c00804 34807584 PMC10149138

[B7] Beebe-Dimmer J. L. Pfeifer J. R. Engle J. S. Schottenfeld D. (2005). The epidemiology of chronic venous insufficiency and varicose veins. Ann. Epidemiol. 15 (3), 175–184. 10.1016/j.annepidem.2004.05.015 15723761

[B8] Cai H. Chen J. Liu J. Zeng M. Ming F. Lu Z. (2017). CRIP1, a novel immune-related protein, activated by *Enterococcus faecalis* in porcine gastrointestinal epithelial cells. Gene 598, 84–96. 10.1016/j.gene.2016.11.009 27836662

[B9] Chang S. L. Huang Y. L. Lee M. C. Hu S. Hsiao Y. C. Chang S. W. (2018). Association of varicose veins with incident venous thromboembolism and peripheral artery disease. Jama 319 (8), 807–817. 10.1001/jama.2018.0246 29486040 PMC5838574

[B10] Cho Y. K. Son Y. Saha A. Kim D. Choi C. Kim M. (2021). STK3/STK4 signalling in adipocytes regulates mitophagy and energy expenditure. Nat. Metab. 3 (3), 428–441. 10.1038/s42255-021-00362-2 33758424

[B11] Conway J. R. Lex A. Gehlenborg N. (2017). UpSetR: an R package for the visualization of intersecting sets and their properties. Bioinformatics 33 (18), 2938–2940. 10.1093/bioinformatics/btx364 28645171 PMC5870712

[B12] Dahlin J. L. Inglese J. Walters M. A. (2015). Mitigating risk in academic preclinical drug discovery. Nat. Rev. Drug Discov. 14 (4), 279–294. 10.1038/nrd4578 25829283 PMC6002840

[B13] Fan F. He Z. Kong L. L. Chen Q. Yuan Q. Zhang S. (2016). Pharmacological targeting of kinases MST1 and MST2 augments tissue repair and regeneration. Sci. Transl. Med. 8 (352), 352ra108. 10.1126/scitranslmed.aaf2304 27535619

[B14] Floyd C. Ni H. Gunaratne R. S. Erban R. Papoian G. A. (2022). On stretching, bending, shearing, and twisting of actin filaments I: Variational models. J. Chem. Theory Comput. 18 (8), 4865–4878. 10.1021/acs.jctc.2c00318 35895330

[B15] Friso S. Pizzolo F. Choi S. W. Guarini P. Castagna A. Ravagnani V. (2008). Epigenetic control of 11 beta-hydroxysteroid dehydrogenase 2 gene promoter is related to human hypertension. Atherosclerosis 199 (2), 323–327. 10.1016/j.atherosclerosis.2007.11.029 18178212

[B16] Fukaya E. Flores A. M. Lindholm D. Gustafsson S. Zanetti D. Ingelsson E. (2018). Clinical and genetic determinants of varicose veins. Circulation 138 (25), 2869–2880. 10.1161/circulationaha.118.035584 30566020 PMC6400474

[B17] Gloviczki P. Comerota A. J. Dalsing M. C. Eklof B. G. Gillespie D. L. Gloviczki M. L. (2011). The care of patients with varicose veins and associated chronic venous diseases: Clinical practice guidelines of the society for vascular surgery and the American venous forum. J. Vasc. Surg. 53, 2S–48s. 10.1016/j.jvs.2011.01.079 21536172

[B18] Gordeuk V. R. Sergueeva A. I. Miasnikova G. Y. Okhotin D. Voloshin Y. Choyke P. L. (2004). Congenital disorder of oxygen sensing: Association of the homozygous Chuvash polycythemia VHL mutation with thrombosis and vascular abnormalities but not tumors. Blood 103 (10), 3924–3932. 10.1182/blood-2003-07-2535 14726398

[B19] Grünberg J. R. Elvin J. Paul A. Hedjazifar S. Hammarstedt A. Smith U. (2018). CCN5/WISP2 and metabolic diseases. J. Cell Commun. Signal. 12 (1), 309–318. 10.1007/s12079-017-0437-z 29247377 PMC5842198

[B20] Grünberg J. R. Hammarstedt A. Hedjazifar S. Smith U. (2014). The novel secreted adipokine WNT1-inducible signaling pathway protein 2 (WISP2) is a mesenchymal cell activator of canonical WNT. J. Biol. Chem. 289 (10), 6899–6907. 10.1074/jbc.M113.511964 24451367 PMC3945351

[B21] Hamdan A. (2012). Management of varicose veins and venous insufficiency. Jama 308 (24), 2612–2621. 10.1001/jama.2012.111352 23268520

[B22] Hammarstedt A. Hedjazifar S. Jenndahl L. Gogg S. Grünberg J. Gustafson B. (2013). WISP2 regulates preadipocyte commitment and PPARγ activation by BMP4. Proc. Natl. Acad. Sci. U. S. A. 110 (7), 2563–2568. 10.1073/pnas.1211255110 23359679 PMC3574913

[B23] Hao Z. Lv D. Ge Y. Shi J. Weijers D. Yu G. (2020). RIdeogram: Drawing SVG graphics to visualize and map genome-wide data on the idiograms. PeerJ. Comput. Sci. 6, e251. 10.7717/peerj-cs.251 33816903 PMC7924719

[B24] Huang T. Zheng Y. Qi Q. Xu M. Ley S. H. Li Y. (2015). DNA methylation variants at HIF3A locus, B-vitamin intake, and long-term weight change: Gene-diet interactions in two U.S. Cohorts. Diabetes 64 (9), 3146–3154. 10.2337/db15-0264 26001398 PMC4542450

[B25] Jiang H. Lun Y. Wu X. Xia Q. Zhang X. Xin S. (2014). Association between the hypomethylation of osteopontin and integrin β3 promoters and vascular smooth muscle cell phenotype switching in great saphenous varicose veins. Int. J. Mol. Sci. 15 (10), 18747–18761. 10.3390/ijms151018747 25329616 PMC4227244

[B26] Kourou K. Exarchos T. P. Exarchos K. P. Karamouzis M. V. Fotiadis D. I. (2015). Machine learning applications in cancer prognosis and prediction. Comput. Struct. Biotechnol. J. 13, 8–17. 10.1016/j.csbj.2014.11.005 25750696 PMC4348437

[B27] Kundu S. Peterson C. L. (2009). Role of chromatin states in transcriptional memory. Biochim. Biophys. Acta 1790 (6), 445–455. 10.1016/j.bbagen.2009.02.009 19236904 PMC2692360

[B28] Langfelder P. Horvath S. (2008). Wgcna: an R package for weighted correlation network analysis. BMC Bioinforma. 9, 559. 10.1186/1471-2105-9-559 PMC2631488 19114008

[B29] Li J. H. Liu S. Zhou H. Qu L. H. Yang J. H. (2014a). starBase v2.0: decoding miRNA-ceRNA, miRNA-ncRNA and protein-RNA interaction networks from large-scale CLIP-Seq data. Nucleic Acids Res. 42, D92–D97. 10.1093/nar/gkt1248 24297251 PMC3964941

[B30] Li Q. Zhu Z. Wang L. Lin Y. Fang H. Lei J. (2021a). Single-cell transcriptome profiling reveals vascular endothelial cell heterogeneity in human skin. Theranostics 11 (13), 6461–6476. 10.7150/thno.54917 33995668 PMC8120211

[B31] Li W. H. Han J. R. Ren P. P. Xie Y. Jiang D. Y. (2021b). Exploration of the mechanism of Zisheng Shenqi decoction against gout arthritis using network pharmacology. Comput. Biol. Chem. 90, 107358. 10.1016/j.compbiolchem.2020.107358 33243703

[B32] Li X. Jiang X. Y. Ge J. Wang J. Chen G. J. Xu L. (2014b). Aberrantly expressed lncRNAs in primary varicose great saphenous veins. PLoS One 9 (1), e86156. 10.1371/journal.pone.0086156 24497937 PMC3908920

[B33] Ling C. Groop L. (2009). Epigenetics: A molecular link between environmental factors and type 2 diabetes. Diabetes 58 (12), 2718–2725. 10.2337/db09-1003 19940235 PMC2780862

[B34] Liu Y. Chen H. Hao J. Li Z. Hou T. Hao H. (2020). Characterization and functional prediction of the microRNAs differentially expressed in a mouse model of concanavalin A-induced autoimmune hepatitis. Int. J. Med. Sci. 17 (15), 2312–2327. 10.7150/ijms.47766 32922197 PMC7484648

[B35] Liu Z. Wang Y. Xu Z. Yuan S. Ou Y. Luo Z. (2021). Analysis of ceRNA networks and identification of potential drug targets for drug-resistant leukemia cell K562/ADR. PeerJ 9, e11429. 10.7717/peerj.11429 34113488 PMC8162247

[B36] Lynch C. J. Liston C. (2018). New machine-learning technologies for computer-aided diagnosis. Nat. Med. 24 (9), 1304–1305. 10.1038/s41591-018-0178-4 30177823

[B37] Mangiola S. Doyle M. A. Papenfuss A. T. (2021). Interfacing Seurat with the R tidy universe. Bioinformatics 37 (22), 4100–4107. 10.1093/bioinformatics/btab404 34028547 PMC9502154

[B38] Otero R. Elías T. Jara L. Trujillo-Santos J. Bertoletti L. Nauffal D. (2013). Factors associated with elevated pulmonary arterial pressure levels on the echocardiographic assessment in patients with prior pulmonary embolism. Thromb. Res. 131 (5), e191–e195. 10.1016/j.thromres.2013.01.034 23466216

[B39] Overington J. P. Al-Lazikani B. Hopkins A. L. (2006). How many drug targets are there? Nat. Rev. Drug Discov. 5 (12), 993–996. 10.1038/nrd2199 17139284

[B40] Qin J. Ke J. Xu J. Wang F. Zhou Y. Jiang Y. (2015). Downregulation of microRNA-132 by DNA hypermethylation is associated with cell invasion in colorectal cancer. Onco. Targets. Ther. 8, 3639–3648. 10.2147/ott.S91560 26675712 PMC4676615

[B41] Qiu X. Huang F. Li Z. Wei X. Wu J. Huang J. (2022). Develop a novel nomogram to predict respiratory failure in acute pancreatitis at early stage. Clin. Lab. 68 (4). 10.7754/Clin.Lab.2021.210826 35443577

[B42] Qu J. Li B. Qiu M. Wang J. Chen Z. Li K. (2022). Discovery of immune-related diagnostic biomarkers and construction of diagnostic model in varies polycystic ovary syndrome. Arch. Gynecol. Obstet. 306 (5), 1607–1615. 10.1007/s00404-022-06686-y 35904610

[B43] Quan Y. Wang Z. Y. Chu X. Y. Zhang H. Y. (2018). Evolutionary and genetic features of drug targets. Med. Res. Rev. 38 (5), 1536–1549. 10.1002/med.21487 29341142

[B44] Raetz J. Wilson M. Collins K. (2019). Varicose veins: Diagnosis and treatment. Am. Fam. Physician 99 (11), 682–688. 31150188

[B45] Raffetto J. D. Ligi D. Maniscalco R. Khalil R. A. Mannello F. (2020). Why venous leg ulcers have difficulty healing: Overview on pathophysiology, clinical consequences, and treatment. J. Clin. Med. 10 (1), E29. 10.3390/jcm10010029 PMC7795034 33374372

[B46] Robin X. Turck N. Hainard A. Tiberti N. Lisacek F. Sanchez J. C. (2011). pROC: an open-source package for R and S+ to analyze and compare ROC curves. BMC Bioinforma. 12, 77. 10.1186/1471-2105-12-77 PMC3068975 21414208

[B47] Robinson M. D. McCarthy D. J. Smyth G. K. (2010). edgeR: a Bioconductor package for differential expression analysis of digital gene expression data. Bioinformatics 26 (1), 139–140. 10.1093/bioinformatics/btp616 19910308 PMC2796818

[B48] Sandru F. Popa A. Paduraru D. N. Filipescu A. Carsote M. Ghemigian A. (2021). Skin anomalies in acromegalic patients (Review of the practical aspects). Exp. Ther. Med. 22 (5), 1330. 10.3892/etm.2021.10765 34630684 PMC8495547

[B49] Scannell J. W. Blanckley A. Boldon H. Warrington B. (2012). Diagnosing the decline in pharmaceutical R&D efficiency. Nat. Rev. Drug Discov. 11 (3), 191–200. 10.1038/nrd3681 22378269

[B50] Schweigert O. Adler J. Längst N. Aïssi D. Duque Escobar J. Tong T. (2021). CRIP1 expression in monocytes related to hypertension. Clin. Sci. 135 (7), 911–924. 10.1042/cs20201372 33782695

[B51] Seok J. (2015). Evidence-based translation for the genomic responses of murine models for the study of human immunity. PLoS One 10 (2), e0118017. 10.1371/journal.pone.0118017 25680113 PMC4332676

[B52] Shannon P. Markiel A. Ozier O. Baliga N. S. Wang J. T. Ramage D. (2003). Cytoscape: A software environment for integrated models of biomolecular interaction networks. Genome Res. 13 (11), 2498–2504. 10.1101/gr.1239303 14597658 PMC403769

[B53] Shi Z. Q. Chen Z. Y. Han Y. Zhu H. Y. Lyu M. D. Zhang H. (2020). WISP2 promotes cell proliferation via targeting ERK and YAP in ovarian cancer cells. J. Ovarian Res. 13 (1), 85. 10.1186/s13048-020-00687-8 32711570 PMC7382796

[B54] Smetanina M. A. Kel A. E. Sevost'ianova K. S. Maiborodin I. V. Shevela A. I. Zolotukhin I. A. (2018). DNA methylation and gene expression profiling reveal MFAP5 as a regulatory driver of extracellular matrix remodeling in varicose vein disease. Epigenomics 10 (8), 1103–1119. 10.2217/epi-2018-0001 30070582

[B55] So P. L. Danielian P. S. (1999). Cloning and expression analysis of a mouse gene related to Drosophila odd-skipped. Mech. Dev. 84 (1-2), 157–160. 10.1016/s0925-4773(99)00058-1 10473132

[B56] Subramanian A. Tamayo P. Mootha V. K. Mukherjee S. Ebert B. L. Gillette M. A. (2005). Gene set enrichment analysis: A knowledge-based approach for interpreting genome-wide expression profiles. Proc. Natl. Acad. Sci. U. S. A. 102 (43), 15545–15550. 10.1073/pnas.0506580102 16199517 PMC1239896

[B57] Tian Y. Morris T. J. Webster A. P. Yang Z. Beck S. Feber A. (2017). ChAMP: Updated methylation analysis pipeline for illumina BeadChips. Bioinformatics 33 (24), 3982–3984. 10.1093/bioinformatics/btx513 28961746 PMC5860089

[B58] Turunen M. P. Aavik E. Ylä-Herttuala S. (2009). Epigenetics and atherosclerosis. Biochim. Biophys. Acta 1790 (9), 886–891. 10.1016/j.bbagen.2009.02.008 19233248

[B59] von Mering C. Huynen M. Jaeggi D. Schmidt S. Bork P. Snel B. (2003). String: A database of predicted functional associations between proteins. Nucleic Acids Res. 31 (1), 258–261. 10.1093/nar/gkg034 12519996 PMC165481

[B60] Wang H. Huang G. (2011). Application of support vector machine in cancer diagnosis. Med. Oncol. 28, S613–S618. 10.1007/s12032-010-9663-4 20842538

[B61] Wang L. Cho K. B. Li Y. Tao G. Xie Z. Guo B. (2019). Long noncoding RNA (lncRNA)-Mediated competing endogenous RNA networks provide novel potential biomarkers and therapeutic targets for colorectal cancer. Int. J. Mol. Sci. 20 (22), E5758. 10.3390/ijms20225758 PMC6888455 31744051

[B62] Wang L. Wang Y. Liu J. Zhao W. (2022). Identification of important genes of keratoconus and construction of the diagnostic model. Genet. Res. 2022, 5878460. 10.1155/2022/5878460 PMC9484959 36160033

[B63] Wang Q. Lan Y. Cho E. S. Maltby K. M. Jiang R. (2005). Odd-skipped related 1 (Odd 1) is an essential regulator of heart and urogenital development. Dev. Biol. 288 (2), 582–594. 10.1016/j.ydbio.2005.09.024 16223478 PMC3869089

[B64] Wang Y. Xu Y. Yang Z. Liu X. Dai Q. (2021). Using recursive feature selection with random forest to improve protein structural class prediction for low-similarity sequences. Comput. Math. Methods Med. 2021, 5529389. 10.1155/2021/5529389 34055035 PMC8123985

[B65] Wilson D. F. (2017). Oxidative phosphorylation: Regulation and role in cellular and tissue metabolism. J. Physiol. 595 (23), 7023–7038. 10.1113/jp273839 29023737 PMC5709332

[B66] Xiao W. Mindrinos M. N. Seok J. Cuschieri J. Cuenca A. G. Gao H. (2011). A genomic storm in critically injured humans. J. Exp. Med. 208 (13), 2581–2590. 10.1084/jem.20111354 22110166 PMC3244029

[B67] Yang S. Wang Y. Chen Y. Dai Q. (2020). Masqc: Next generation sequencing assists third generation sequencing for quality control in N6-methyladenine DNA identification. Front. Genet. 11, 269. 10.3389/fgene.2020.00269 32269589 PMC7109398

[B68] Yang Z. Yi W. Tao J. Liu X. Zhang M. Q. Chen G. (2022). HPVMD-C: A disease-based mutation database of human papillomavirus in China. Database. Oxford. 10.1093/database/baac018 PMC9216535 35348640

[B69] Yu G. Wang L. G. Han Y. He Q. Y. (2012). clusterProfiler: an R package for comparing biological themes among gene clusters. Omics 16 (5), 284–287. 10.1089/omi.2011.0118 22455463 PMC3339379

[B70] Zhang F. Jiang Z. (2020). Downregulation of OSR1 promotes colon adenocarcinoma progression via FAK-mediated akt and MAPK signaling. Onco. Targets. Ther. 13, 3489–3500. 10.2147/ott.S242386 32425550 PMC7191353

[B71] Zhang Z. Wang C. (2022). Exploring key genes and pathways of cardiac hypertrophy based on bioinformatics. Dis. Markers 2022, 2081590. 10.1155/2022/2081590 36046382 PMC9423995

