# Supplementary figures and images for: Comprehensive bioinformatics analysis reveals biomarkers of DNA methylation-related genes in varicose veins
Source: Front Genet. 2022 Nov 25;13:1013803. doi: 10.3389/fgene.2022.1013803 (PMC9732536; doi:10.3389/fgene.2022.1013803)

Supplementary Material


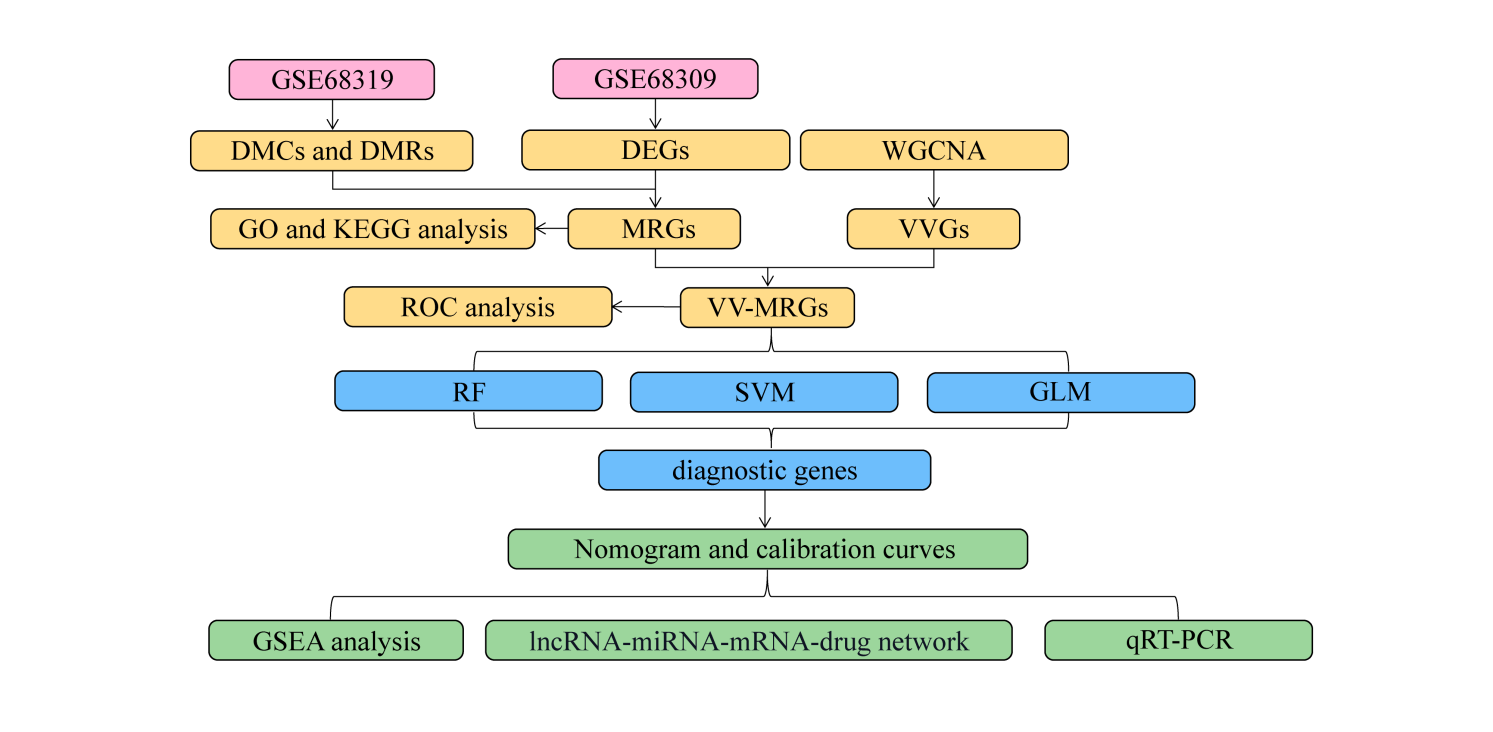


**Supplementary Figure 1.** The whole flowchart of the study.

Supplement: Supplementary file 1 [file DataSheet1.ZIP › Supplementary Material (2022.10.31)/Supplementary Figure 1.docx]
